# Design, Characteristics, and Implementation of the Financial Support for Low-Income Patients with Heart Failure Trial

**DOI:** 10.1016/j.jacadv.2026.102698

**Published:** 2026-03-16

**Authors:** Syed K. Rizvi, Neil Keshvani, Anand K. Jain, Myriam Bustillo-Rubio, Matthew W. Segar, Juan David Coellar, James W. Miller, Thomas J. Wang, Eric D. Peterson, Ambarish Pandey

**Affiliations:** a Division of Cardiology, Department of Medicine, University of Texas Southwestern Medical Center, Dallas, Texas, USA; b Baylor Scott and White Research Institute, Dallas, Texas and Baylor Scott and White The Heart Hospital, Plano, Texas, USA; c Department of Cardiology, Texas Heart Institute, Houston, Texas, USA; d Department of Medicine, Texas Health Presbyterian Hospital, Dallas, Texas, USA; e University of Michigan School of Medicine, Ann Arbor, Michigan, USA

**Keywords:** adherence, financial support, heart failure, quality of life, social determinants of health

## Abstract

**Background:**

Patients with heart failure (HF) with socioeconomic hardship have poor medication adherence and worse patient outcomes. Whether an early postdischarge financial support intervention can improve care behaviors and outcomes among socioeconomically disadvantaged populations is unclear.

**Objectives:**

To describe the design, socioeconomic eligibility framework, therapeutic drug monitoring methodology, and baseline characteristics of the FUND-HF trial, a pilot randomized controlled trial evaluating the feasibility and effect of early postdischarge financial support among low-income patients with HF with reduced ejection fraction. The primary clinical outcomes are reported in a companion Brief Report.

**Methods:**

In this pilot randomized controlled trial (NCT05928026), adults hospitalized for acute HF with income <130% federal poverty limit and difficulty paying bills with ≥1 additional socioeconomic need were randomized within 14 days of discharge 1:1 to early financial support ($500 debit card) vs control ($500 at 30-day follow-up). Key outcomes were medication adherence assessed by therapeutic drug monitoring, expressed as percent of tested cardiac drugs with detectable serum levels and categorized as complete/partial/none, assessed overall and for HF guideline-directed medical therapy with available assays (metoprolol, spironolactone) and the Kansas City Cardiomyopathy Questionnaire at 1 month. Data on 30-day all-cause hospitalizations and outpatient visit attendance were also collected.

**Results:**

Of the 153 randomized participants (n = 76 financial support; n = 77 control; mean 53 y, 76% male, 73% Black; mean ejection fraction 26 ± 9%), 140 completed 1-month follow-up. Participants had median monthly income of $600 (IQR 0-1179), with 76% reporting cost-related medication non-adherence at baseline. On average, participants had 5 identified socioeconomic domain needs. Housing instability was reported by 50% of participants, while 85% reported food insecurity and 65% reported transportation barriers. 81% of participants were unemployed or retired. Baseline characteristics were well balanced between groups except for numerically higher median monthly income and lower mineralocorticoid receptor antagonist/sodium-glucose cotransporter-2 inhibitor prescription in the financial support group.

**Conclusions:**

The FUND-HF pilot trial successfully enrolled a deeply socioeconomically disadvantaged HF with reduced ejection fraction population using a multidomain social determinants of health eligibility framework with comprehensive socioeconomic phenotyping across multiple domains. This pilot design and implementation framework provides a practical template for future larger trials targeting economic barriers in cardiovascular populations. The clinical findings of this pilot trial are presented in the companion report.

Heart failure (HF) affects 6.7 million Americans and is a leading cause of hospitalization in the United States.^1^, ^2^, ^3^ The postdischarge transition period represents a critical vulnerability window, with high rates of readmission and mortality that increase with each successive hospitalization.^4^, ^5^, ^6^, ^7^ This risk of adverse postdischarge outcomes is disproportionately higher among socioeconomically disadvantaged patients, including racial and ethnic minorities, who experience significantly worse postdischarge outcomes.^8^ These disparities are due to a high burden of adverse social determinants of health (SDOH), including inadequate access to evidence-based therapies; limited health care follow-up; and competing priorities for basic necessities such as housing, food, and transportation.^9^, ^10^, ^11^, ^12^, ^13^ Addressing these upstream SDOH represents a critical but underexplored approach to reducing cardiovascular inequities.

Current interventions to improve postdischarge HF outcomes have primarily focused on health care system optimization through transitional care programs, including multidisciplinary teams, care coordination, and patient navigation.^14^ Recent evidence from multicenter trials such as CONNECT-HF and PACT-HF did not demonstrate a significant reduction in readmissions or mortality despite intensive care coordination efforts.^15^^,^^16^ These findings suggest that optimizing health care navigation may be insufficient when fundamental economic barriers prevent patients from affording medications, transportation, or addressing competing survival priorities.

Prior financial interventions in cardiovascular populations have primarily targeted single domains of cost-related nonadherence, such as eliminating medication copayments or providing vouchers for specific drug classes, with improvements in adherence but limited effects on clinical outcomes.^17^^,^^18^ Financial incentives have also been evaluated to promote cardiac rehabilitation attendance among low-socioeconomic status patients.^19^ However, these approaches addressed isolated barriers rather than the broader economic conditions that drive nonadherence and adverse outcomes in socioeconomically disadvantaged populations. This represents an important knowledge gap, as socioeconomic barriers to postdischarge recovery are multifaceted, and addressing these upstream SDOH may require a fundamentally different approach.

Direct, unrestricted financial support may enable patients to prioritize their most pressing needs rather than health care providers determining which single barrier to address. However, unrestricted financial support interventions for patients with acute conditions, such as HF, during high-risk postdischarge periods remain largely unexplored.^20^ We therefore conducted a pilot randomized controlled trial (RCT) of early postdischarge financial support among patients with HF with reduced ejection fraction (HFrEF) and substantial adverse SDOH, with the primary clinical outcomes reported in the accompanying brief report in *JACC*.^21^

Given the novelty of this intervention in the postdischarge HF setting, this study was designed as a pilot trial with several key objectives: 1) to assess the feasibility of delivering a financial support intervention during the vulnerable postdischarge transition period; 2) to identify which outcomes are most likely to be affected by the intervention and have biologic plausibility for improvement with financial support; 3) to generate preliminary treatment effect estimates that could be used to power a larger, definitive trial; and 4) to evaluate whether there are unintended adverse consequences of the intervention, including increased recreational drug use or adverse psychological stress among the control arm participants. In this article, we provide a detailed description of the trial design, operationalization frameworks, and baseline characteristics with practical considerations for implementing financial support interventions in cardiovascular populations.

## Methods

### Study design and population

In this pilot RCT, adults aged ≥18 years with HFrEF were recruited within 2 weeks of hospitalization for HF at Parkland Memorial Hospital, a large, urban, safety-net hospital in Dallas, Texas. Parkland Memorial Hospital is the primary public hospital for Dallas County, serving as a critical access point for a predominantly uninsured and underinsured population. The hospital is supported by Dallas County taxpayer funding and operates as part of an integrated health system that includes a network of community-based clinics providing primary and specialty care. The insurance mix at Parkland includes a substantial proportion of uninsured patients, those covered by the county’s indigent care program (Parkland Financial Assistance), Medicaid, and Medicare, with a smaller proportion of commercially insured patients. Notably, Parkland Financial Assistance provides prescription medications through its own pharmacy system, which substantially reduces but does not eliminate medication costs for enrolled patients. This study was approved by the University of Texas Southwestern Medical Center Institutional Review Board (STU-2022-1033) and was registered at ClinicalTrials.gov (NCT05928026). All participants provided written informed consent before enrollment.

Potentially eligible patients were identified through the electronic health records–based registry of hospitalized HFrEF patients. The HFrEF registry included all patients with a left ventricular ejection fraction ≤40% based on the most recent echocardiogram. Patients deemed eligible on initial review of clinical data in the electronic medical records were approached close to their discharge from the acute hospital stay to complete an in-hospital eligibility screening assessment to assess socioeconomic distress across multiple domains, including employment insecurity, housing instability, household crowding, history of cost-related medication nonadherence, food insecurity, transportation barriers, low social support, interpersonal violence, or history of major discrimination (Supplemental Table 1). Each domain was dichotomized to identify individuals with adverse SDOH. Participants were eligible if they had household income below 130% of the Federal Poverty Level, difficulty paying monthly bills, and had at least 1 additional adverse SDOH (Table 1). Exclusion criteria included residence outside the hospital’s primary safety-net service area (Dallas County), incarceration, non-English as primary language, systolic blood pressure <90 mm Hg at screening, receipt of advanced HF therapies, or no medications prescribed at discharge that were amenable to therapeutic drug monitoring (TDM).

**Table 1:** Inclusion and Exclusion Criteria

Inclusion criteria
• Age ≥18 years • Hospitalization for heart failure defined as acute worsening of heart failure symptoms necessitating inpatient hospitalization requiring treatment with intravenous diuretics • Last documented echocardiogram with an EF ≤ 40% • Eligible to receive at least 1 of the following medications at discharge for drug level assessment: metoprolol, spironolactone, furosemide, amlodipine, nifedipine, hydralazine, or prazosin • Annual household income ≤130% Federal Poverty Limit of the year off enrollment (From 2023-$14,580 USD to 2025-$15,650 USD) • Self-reported difficulty paying usual bills • At least 1 additional socioeconomic need: ○ Cost-related nonadherence ○ Food insecurity ○ Housing instability (including homelessness) ○ Transportation barrier ○ Unemployment defined as either unemployment or retirement ○ Household crowding: person/room ratio >1 ○ Low social support ○ Interpersonal violence ○ History of major discrimination
Exclusion criteria
• Not prescribed or not able to tolerate a medication available for therapeutic drug monitoring • Unable or unwilling to complete 1-month follow-up • Incarcerated • Primary residence outside of primary coverage area for safety-net hospital (Dallas County) • Legally blind • Systolic blood pressure <90 mmHg at screening • Under consideration for or recipient of advanced heart failure therapies (LVAD, heart transplant) • Inability to provide written consent

EF = ejection fraction; LVAD = left ventricular assist device; USD = U.S. dollars.

### Randomization and study intervention

Eligible participants who consented to participate in the trial were invited for a baseline visit within 14 days of discharge (Central Illustration). Participants were randomized in a 1:1 ratio through REDCap to receive either early financial support or control (receiving delayed financial support). The randomization sequence was computer-generated in an open-label design. Participants randomized to the early financial support group received $500 on a UT Southwestern ClinCard immediately at randomization. The entire amount was provided as a single disbursement without restrictions on spending categories or usage. Participants could use the card for purchases with debit card functionality or withdraw funds as cash. No guidance was provided regarding fund utilization except that funds were intended to support their postdischarge recovery. The type of spending (eg medication copayments vs retail purchases vs other) was available for a subset of participants. Participants in the control group received usual postdischarge care without the early financial support. Control group participants received an equivalent payment upon completion of the 1-month follow-up visit.

**Central Illustration:**
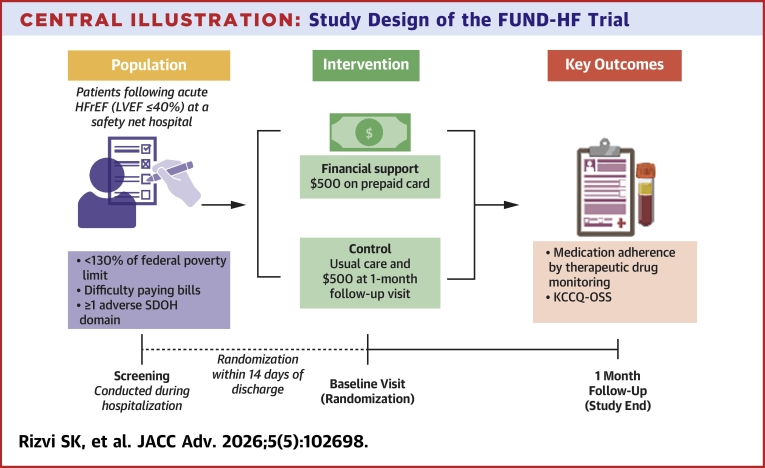
Study Design of the FUND-HF Trial Participants with acute HFrEF and socioeconomic hardship were screened during hospitalization at a safety-net hospital. Eligible participants, defined by income <130% of the federal poverty level, difficulty paying bills, and ≥1 additional adverse SDOH domain, were randomized within 14 days of discharge to receive $500 in unrestricted financial support on a prepaid card or control (with usual care and $500 provided at the 1-month follow-up visit). Key outcomes assessed at 1 month included objective medication adherence by therapeutic drug monitoring and health-related quality of life by KCCQ-OSS. HFrEF = heart failure with reduced ejection fraction; KCCQ-OSS = Kansas City Cardiomyopathy Questionnaire Overall Summary Score; LVEF = left ventricular ejection fraction; SDOH = social determinants of health.

### Follow-up

Participants completed a follow-up visit at 1 month postrandomization. Blood samples for TDM were collected at the follow-up visit and sent for analysis by liquid chromatography tandem mass spectrometry at NMS Labs, Inc. Urine samples were obtained at the follow-up visit to assess for substance use during the study period. At the follow-up visit, participants completed psychosocial assessments and reported spending patterns with standardized questionnaires administered by trained research personnel. Participants had the option to voluntarily share financial transaction records from their debit cards for descriptive spending pattern analysis. Clinical events were captured through comprehensive medical record review of the integrated health system electronic medical record and participant report during the follow-up period.

### Study outcomes

Key outcomes were medication adherence by TDM and health-related quality of life (HRQOL), assessed by the Kansas City Cardiomyopathy Questionnaire-12 Overall Summary Score (KCCQ-OSS) at 1-month follow-up.

#### Medication adherence by TDM

Objective medication adherence was assessed by measuring serum levels of cardiac medications prescribed at discharge that were amenable to TDM at the 1-month follow-up visit. These medications included metoprolol, spironolactone, furosemide, amlodipine, nifedipine, hydralazine, and prazosin. Adherence was evaluated both as the proportion of tested medications with detectable levels and categorically as nonadherent (no tested medications detected), partially adherent (some but not all tested medications detected), or completely adherent (all tested medications detected).

#### Patient-reported outcomes

The KCCQ-OSS was calculated as the average of 4 subdomains: physical limitation, symptom frequency, quality of life, and social limitation, with scores ranging from 0 to 100, where higher scores indicate better HRQOL.^22^ Additional patient-reported outcomes included medication adherence assessed by the Morisky Medication Adherence Scale, 8-item (MMAS-8) and psychosocial distress evaluated using the Kessler Psychological Distress Scale (K10).^23^^,^^24^

#### Clinical outcomes and health care utilization

Health care utilization outcomes included outpatient follow-up visit attendance, which was calculated as the ratio of completed to scheduled clinic visits during the follow-up period. The prespecified clinical outcome was all-cause hospitalization at 30 days. HF rehospitalizations were assessed as an exploratory post hoc analysis given their clinical relevance and were classified based on a primary or first secondary diagnosis of HF documented in the physician discharge summary, consistent with prior epidemiologic studies.^25^

#### Safety outcomes

Safety outcomes included urine drug screening positivity and perceived stress measured with the Perceived Stress Scale (PSS-10) at 1-month follow-up.^26^

### Statistical analysis

As this was an exploratory pilot study, sample size was determined pragmatically based on study timeline and recruitment feasibility rather than formal power calculations, resulting in enrollment of 153 participants. To inform the design of a subsequent larger trial, we conducted a post hoc power analysis using observed effect sizes and variability. Using the model-based SE for objective medication adherence derived from the final analytic sample (66 participants in the intervention arm and 74 in the control arm; 140 total with complete follow-up), we estimated that the study had 80% power at a 2-sided α of 0.05 to detect an absolute between-group difference of approximately 0.20 in mean proportional medication adherence by serum drug levels. In addition, using the observed baseline KCCQ-OSS SD (21.7 points) and the same analytic sample, we estimated that the study had 80% power at 2-sided α of 0.05 to detect a between-group difference of 10.3 points in KCCQ-OSS. These estimates are intended to inform sample size calculations for future trials rather than to justify the design or findings of the current analysis.

Baseline characteristics were summarized across study groups and presented using mean (SD) or median (IQR) for continuous variables and as count (percentage) for categorical variables. Key study outcomes were analyzed per-protocol, including only participants who completed the 1-month follow-up visit. Health care utilization and clinical outcomes were analyzed using intention-to-treat principles regardless of follow-up completion status.

Medication adherence at 1-month follow-up was assessed as the proportion of all tested medications with detectable serum levels (number of tested medications with detectable levels ÷ total medications tested). Adherence was also assessed categorically, and participants were identified as nonadherent (proportion = 0), partially adherent (proportion >0 to <1.0), or completely adherent (proportion = 1.0). The effect of financial support intervention (vs control group) on medication adherence was assessed using separate statistical models. For the proportion of tested medications with detectable levels, a binomial generalized linear model was used, with the number of detected drugs modeled relative to the total number tested per participant. Estimated marginal means (expressed as proportions) with 95% CIs were derived for each treatment group, and between-group differences were reported as absolute difference in proportions. For categorical adherence outcomes, modified Poisson regression with robust SEs was used to estimate risk ratios and 95% CIs comparing each adherence category (complete or partial) to the nonadherent group as the reference. Separate models were constructed for all tested medications and for guideline-directed medical therapy (GDMT) specifically (metoprolol and spironolactone).

KCCQ-OSS at 1 month was analyzed using analysis of covariance through linear regression with baseline KCCQ-OSS as a covariate, with results presented as between-group differences in adjusted least-squares means and 95% CIs. The additional patient-reported outcomes including MMAS-8, K10, and PSS-10 were analyzed similarly.

Outpatient clinic attendance was evaluated by calculating completion percentages for primary care physician, cardiology, other specialty, and transitional care outpatient visits. Percentages of participants who completed all scheduled visits and those who completed no visits were assessed and compared between groups using logistic regression to determine ORs and 95% CIs. All-cause and HF hospitalizations at 30 days were analyzed as event rates per 100 person-months using negative binomial regression to accommodate zero-inflation and right-skewed distribution of count data, with results presented as rate ratios and 95% CIs. A person-time offset term was included to account for potential unequal exposure time.

We also evaluated the effect of the financial support intervention on TDM-based adherence and HRQOL as described previously with additional adjustment for log-transformed baseline monthly income to account for observed income differences between groups at baseline. In addition, stratified treatment effects were estimated by adverse SDOH burden (above vs below median of 5 domains) (Supplemental Table 1) and baseline income (above vs below median of $600). Interaction terms between treatment assignment (financial support vs control) and each stratifying characteristic were tested to assess differential treatment effect modification.

Missing data were handled using complete case analysis. As a sensitivity analysis to evaluate the robustness of primary study findings due to differential missingness across study arms, reference-based multiple imputation was conducted using 20 imputed datasets pooled using Rubin rule.^27^ Missing outcomes for participants in the financial support group (n = 10) were imputed using the control group's outcome distribution, conservatively assuming no treatment effect among those lost to follow-up, whereas missing outcomes for control group (n = 3) participants were imputed under a missing-at-random assumption using their own group's distribution. All statistical tests were 2-sided with significance set at α = 0.05. Analyses were performed using R (version 4.2.2; R Foundation for Statistical Computing).

## Results

### Eligibility and enrollment

**Figure 1:**
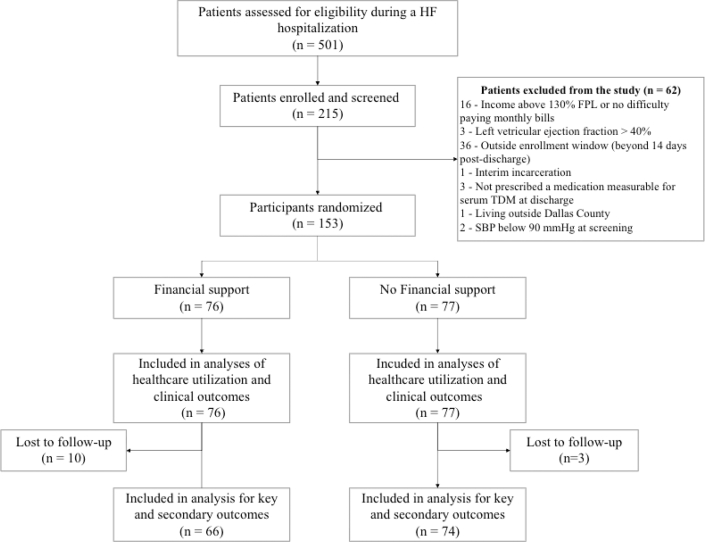
The CONSORT Diagram Flow of participants through screening, randomization, and follow-up in the FUND-HF trial. FPL = federal poverty limit; HF = heart failure; SBP = systolic blood pressure; TDM = therapeutic drug monitoring.

Between June 2023 and February 2025, 215 participants were screened and underwent in-hospital baseline eligibility assessments (Figure 1). Of these, 153 eligible participants were enrolled and randomized to receive either financial support (n = 76) or control (n = 77). A total of 140 participants completed the 1-month follow-up. The proportion completing 1-month follow-up was 91.5% overall, with greater follow-up in the control group (96.1%) vs the financial support group (86.8%). One participant in the control group was switched from furosemide to torsemide before the baseline visit, leaving no medications amenable to TDM; this participant was excluded from TDM analyses. Participants lost to follow-up (n = 13) were similar to those who completed follow-up with respect to baseline demographics, clinical characteristics, socioeconomic burden, and medication prescriptions at discharge (Supplemental Table 2).

### Baseline characteristics and burden of socioeconomic needs

At baseline, the mean (SD) age was 52.8 (11.0) years, 76% were males, 73% identified as Black race, and more than half were uninsured (54%) (Table 2). 35% of participants (83% uninsured and 17% with some insurance) were additionally enrolled in Parkland Hospital’s county indigent health program. Participants were enrolled a median of 6 days (IQR: 4,8) after discharge from index hospitalization. On average, participants had 5 identified SDOH domain needs. The $500 financial support represented 83% of the median monthly income. 81% of participants were unemployed or retired. Housing instability was reported by 50% of participants, whereas 85% reported food insecurity and 65% reported transportation barriers. Most participants (78%) had experienced prior major discrimination, and 45% reported inadequate social support. 76% of participants reported cost-related medication nonadherence.

**Table 2:** Baseline Characteristics

	Total (N = 153)	Financial Support (n = 76)	Control (n = 77)
Age, y	52.8 (11.0)	53.1 (10.8)	52.5 (11.3)
Male	116 (75.8)	59 (77.6)	57 (74.0)
Race/ethnicity			
Non-Hispanic White	16 (10.5)	10 (13.2)	6 (7.8)
Hispanic White	23 (15.0)	14 (18.4)	9 (11.7)
Black	111 (72.5)	49 (64.5)	62 (80.5)
Other	3 (2.0)	3 (3.9)	0 (0.0)
County indigent health program	54 (35.3)	26 (34.2)	28 (36.4)
Insurance			
Uninsured with county indigent support	45 (29.4)	22 (28.9)	23 (29.9)
Uninsured without county indigent support	38 (24.8)	17 (22.4)	21 (27.3)
Medicare/Medicaid/dual eligible	48 (31.4)	25 (32.9)	23 (29.9)
Marketplace/private	22 (14.4)	12 (15.8)	10 (13.0)
Clinical characteristics			
Hypertension	138 (90.2)	71 (93.4)	67 (87.0)
Diabetes	61 (39.9)	31 (40.8)	30 (39.0)
Hyperlipidemia	81 (52.9)	39 (51.3)	42 (54.5)
CVA/TIA	19 (12.4)	12 (15.8)	7 (9.1)
Coronary artery disease	44 (28.8)	24 (31.6)	20 (26.0)
Mental health disease	28 (18.3)	15 (19.7)	13 (16.9)
Atrial fibrillation/flutter	58 (37.9)	30 (39.5)	28 (36.4)
Substance use disorder	31 (20.3)	17 (22.4)	14 (18.2)
NYHA functional class III	71 (46.4)	35 (46.1)	36 (46.8)
Non-ischemic HF etiology	124 (81.0)	61 (80.3)	63 (81.8)
New HF diagnosis	17 (11.1)	9 (11.8)	8 (10.4)
LVEF	25.0 [20.0, 33.0]	25.5 [20.0, 34.0]	25.0 [20.0, 33.0]
Implantable cardioverter defibrillator	46 (30.1)	23 (30.3)	23 (29.9)
BMI, kg/m^2^	30.6 [25.7, 39.9]	30.1 [25.5, 38.8]	31.7 [26.0, 40.5]
SBP, mm Hg	119 [107, 137]	118 [109, 134]	123 [104, 138]
Discharge eGFR, mL/min/1.73 m^2^	59.9 (20.4)	61.7 (22.3)	58.1 (18.2)
Admission NT-proBNP, pg/mL	3,657.0 [2,208.0, 7,357.0]	3,648.0 [1930.5, 7,211.5]	3,775.0 [2,593.0, 7,574.0]
Social needs and eligibility			
Days from randomization to discharge	6.0 [4.0, 8.0]	6.0 [4.0, 8.0]	6.0 [4.0, 8.0]
Self-reported monthly income, $	600 [0, 1,179]	840.5 [0, 1,300]	400 [0, 1,000]
Self-reported monthly income of $0	60 (39.2)	28 (36.8)	32 (41.6)
Domains of social need^a^			
Unemployment	124 (81.0)	59 (77.6)	65 (84.4)
Housing instability	76 (49.7)	35 (46.1)	41 (53.2)
Household crowding	41 (26.8)	21 (27.6)	20 (26.0)
Cost-related medication nonadherence	116 (75.8)	56 (73.7)	60 (77.9)
Food insecurity	130 (85.0)	61 (80.3)	69 (89.6)
Transportation barriers	99 (64.7)	46 (60.5)	53 (68.8)
Low social support	69 (45.1)	31 (40.8)	38 (49.4)
Interpersonal violence	15 (9.8)	7 (9.2)	8 (10.4)
History of major discrimination	119 (77.8)	59 (77.6)	60 (77.9)
Total	5.0 [4.0, 7.0]	5.0 [4.0, 6.0]	5.0 [4.0, 7.0]
HF medication utilization			
Beta-blocker	131 (85.6)	68 (89.5)	63 (81.8)
RASi	133 (86.9)	67 (88.2)	66 (85.7)
MRA	126 (82.4)	57 (75.0)	69 (89.6)
SGLT2i	123 (80.4)	56 (73.7)	67 (87.0)
Quadruple GDMT	88 (57.5)	40 (52.6)	48 (62.3)
Not prescribed any GDMT	0 (0.0)	0 (0.0)	0 (0.0)
Metoprolol	91 (59.5)	45 (59.2)	46 (59.7)
Spironolactone	123 (80.4)	57 (75.0)	66 (85.7)
Furosemide	68 (44.4)	39 (51.3)	29 (37.7)
Amlodipine	8 (5.2)	4 (5.3)	4 (5.2)
Hydralazine	12 (7.8)	6 (7.9)	6 (7.8)
Nifedipine	0 (0.0)	0 (0.0)	0 (0.0)
Prazosin	1 (0.7)	0 (0.0)	1 (1.3)
Prescribed a testable GDMT (metoprolol or spironolactone)	143 (93.5)	70 (92.1)	73 (94.8)
Number of medications amenable to TDM per participant	2 [1, 2]	2 [1, 2]	2 [1, 3]
Proportion of prescribed cardiovascular medications per participant amenable to TDM	0.34 (0.14)	0.34 (0.14)	0.33 (0.14)
Questionnaires			
KCCQ-OSS, numeric	39.1 [24.0, 56.2]	39.3 [26.0, 62.6]	39.1 [23.4, 50.0]
MMAS-8, numeric	5.8 [4.0, 7.0]	5.6 [3.8, 7.0]	5.8 [4.5, 7.0]
MMAS-8, categorical			
Low adherence	89 (58.2)	45 (59.2)	44 (57.1)
Medium/high adherence	64 (41.8)	31 (40.8)	33 (42.9)
Self-reported spending, $			
Health care	2.5 [0.0, 85.2]	2.5 [0.0, 90.0]	5.0 [0.0, 81.2]
Bills/essentials	447.5 [176.2, 837.8]	485.0 [223.5, 866.2]	417.5 [153.8, 816.8]
Miscellaneous	85.0 [0.0, 206.2]	90.0 [0.0, 206.2]	85.0 [0.0, 205.0]

Values shown are n (%), mean (SD), or median [IQR].

Low adherence defined as MMAS-8 score <6; Medium adherence = 6 to <8; High adherence = 8.

BMI = body mass index; CVA/TIA = cerebrovascular accident/transient ischemic attack; eGFR = estimated glomerular filtration rate; GDMT = guideline-directed medical therapy; HF = heart failure; KCCQ-OSS = Kansas City Cardiomyopathy Questionnaire Overall Summary Score; LVEF = left ventricular ejection fraction; MMAS-8 = Morisky Medication Adherence Scale, 8-item; MRA = mineralocorticoid receptor antagonist; NT-proBNP = N-terminal pro B-type Natriuretic Peptide; RASi = renin-angiotensin system inhibitor; SBP = systolic blood pressure; SGLT2i = sodium-glucose cotransporter-2 inhibitor; TDM = therapeutic drug monitoring.

a Domains of social need assessed using the following: unemployment defined as unemployed or retired, housing stability defined as self-reported concern about current or future housing, household crowding defined as ratio of residents to rooms >1, cost-related medication adherence as at least one affirmative answer to the 4 cost-related medication nonadherence questions adapted from the Medicare Current Beneficiary Survey (MCBS); food insecurity defined as raw score ≥2 using the U.S. Household Food Security Survey Module: Six-Item Short Form; transportation barriers defined as self-reported difficulty with transportation to access medical care; low social support defined as <18 on ENRICHD Social Support Instrument; interpersonal violence defined as ≥10 on Hurt Insult Threaten Scream (HITS) screening tool; history of major discrimination defined as at least one affirmative response to the Everyday Discrimination Scale or Major Experiences of Discrimination Questionnaire.

Most participants were NYHA functional class II (54%) with a median (IQR) left ventricular ejection fraction of 25% (20%, 33%) and a median (IQR) KCCQ-OSS of 39.1 (24.0, 56.3). Comorbidities were common, including hypertension (90%) and diabetes (39%). Of cardiovascular medications prescribed at discharge, approximately 34% were amenable to TDM, with a median of 2 testable medications per participant. 58% of participants received quadruple GDMT. Among testable GDMT, metoprolol and spironolactone were prescribed in 60% and 80% of participants, respectively.

At baseline, participants in the financial support group (vs control) were less likely to be of Black race and had higher median monthly income compared with the control group ($840 vs $400), although the proportion reporting $0 monthly income was similar between groups (37% vs 42%). Prescription of mineralocorticoid receptor antagonist (75% vs 90%) and sodium-glucose cotransporter-2 inhibitor (74% vs 87%) at discharge was lower in the financial support group compared with the control group. Baseline characteristics were otherwise well balanced between groups.

### Effects of the financial support intervention on adherence and HRQOL

The distributions of individual serum drug concentrations assessed at 1-month across randomization groups are shown in Supplemental Figure 1. The primary effects of the intervention on adherence, patient-reported measures, and health care utilization/clinical outcomes are reported in the companion piece published in *JACC*.^21^ Here, we report the results of several sensitivity analyses to demonstrate the robustness of our findings.

In sensitivity analyses adjusting for baseline self-reported income, the financial support group demonstrated significantly higher adherence to all tested medications compared with control (estimated absolute difference of financial support vs control [95% CI]: 0.21 [0.09-0.33]; *P* = 0.001), with consistent findings for adherence to GDMT (difference: 0.22 [0.09-0.35]; *P* = 0.001) (Table 3). KCCQ-OSS did not differ between groups after income adjustment (difference: 0.7 [-5.9 to 7.2]; *P* = 0.84). Results were also consistent in reference-based multiple imputation analyses for adherence to all tested medications (difference: 0.23 [0.11-0.34]; *P* < 0.001) and GDMT (difference: 0.23 [0.10-0.35]; *P* < 0.001) without difference in KCCQ-OSS between groups (least-squares mean difference: −0.4 [-6.8 to 6.0]; *P* = 0.90).

**Table 3:** Sensitivity Analyses for Treatment Effects on Medication Adherence and Quality of Life at 1 Month Using Income-Adjusted and Reference-Based Multiple Imputation Models

	Adjusted Difference (95% CI)	*P* Value
Adjustment for baseline income^a^		
Medication adherence		
All medications		
Proportional adherence, mean (95% CI)	0.21 (0.09-0.33)	0.001
GDMT only		
Proportional adherence, mean (95% CI)	0.22 (0.09-0.35)	0.001
Health-related quality of life		
KCCQ-OSS, LSM difference	0.7 (−5.9 to 7.2)	0.84
Reference-based imputation^b^		
Medication adherence		
All medications		
Proportional adherence, mean (95% CI)	0.23 (0.11-0.34)	<0.001
GDMT only		
Proportional adherence, mean (95% CI)	0.23 (0.10-0.35)	<0.001
Health-related quality of life		
KCCQ-OSS, LSM difference	−0.4 (−6.8 to 6.0)	0.90

Proportional adherence estimated using binomial regression with estimated marginal means, expressed as absolute differences in proportions (0-1). KCCQ-OSS analyzed using linear regression adjusted for baseline KCCQ-OSS. TDM assessed 7 cardiovascular medications: metoprolol, spironolactone, furosemide, amlodipine, nifedipine, hydralazine, prazosin. GDMT adherence outcomes restricted to metoprolol and spironolactone.

LSM = least-squares mean; other abbreviations as in Table 2.

a Models adjusted for treatment arm and log-transformed baseline monthly income.

b Reference-based multiple imputation used 20 imputations pooled using Rubin's rules; missing outcomes for financial support group participants were imputed using the control group's outcome distribution, while missing control group outcomes were imputed under a missing-at-random assumption.

The effect of financial support vs control on medication adherence or KCCQ-OSS was not modified by baseline income (p_interaction_ treatment × income >0.1 for all outcomes). The treatment effect on adherence to all tested medications was similar between income strata (estimated absolute difference of financial support vs control [95% CI]: Below median income: 0.22 [0.05-0.39]; *P* = 0.012; above median income: 0.19 [0.02-0.35]; *P* = 0.025) (Supplemental Table 3). Adherence to GDMT and KCCQ-OSS at 1 month were similar across baseline income strata. There was a trend toward treatment effect modification by adverse SDOH burden for adherence to all tested medications (p_interaction_ treatment × SDOH burden = 0.053). The treatment effect was significant among participants with greater SDOH burden (difference: 0.35 [0.17-0.53]; *P* < 0.001) but not among those with lower SDOH burden (difference: 0.09 [-0.06 to 0.24]; *P* = 0.24). SDOH burden also significantly modified the treatment effect on adherence to GDMT (p_interaction_ treatment × SDOH burden = 0.016). Participants with greater SDOH burden demonstrated a significant improvement in adherence to GDMT (difference: 0.42 [0.23-0.60]; *P* < 0.001) whereas those with lower burden of SDOH had similar adherence to GDMT between groups (difference: 0.06 [-0.11 to 0.23]; *P* = 0.47). KCCQ-OSS at 1 month was similar across strata of SDOH burden without treatment effect modification (p_interaction_ treatment × SDOH burden = 0.51).

### Health care utilization and clinical outcomes

The percent of completed outpatient follow-up visits were similar between the financial support vs control groups (60% vs 57%; *P* = 0.72), without significant differences in primary care, transitional care, cardiology, or other specialty visits (Supplemental Table 4). HF hospitalization rates were 21.8 vs 30.5 events per 100 person-months in the financial support and control groups, respectively (rate ratio: 0.71; 95% CI: 0.38-1.33; *P* = 0.29) (Table 4).

**Table 4:** Additional Study Outcomes at 1 Month

	Financial Support (n = 66)	Control (n = 74)	Difference, Rate Ratio, or Risk Ratio (95% CI)	*P* Value
Clinical outcomes				
HF hospitalization rate	21.8 events per 100 PM	30.5 events per 100 PM	Rate Ratio: 0.71 (0.38, 1.33)	0.29
Self-reported adherence, stress, and psychological distress				
MMAS-8, LSM (95% CI)	6.2 (5.8, 6.6)	5.8 (5.5, 6.2)	0.4 (−0.1, 0.9)	0.15
PSS-10, LSM (95% CI)	15.3 (13.6, 16.9)	16.5 (14.9, 18.1)	−1.3 (−3.6, 1.1)	0.29
K10, LSM (95% CI)	20.7 (19.0, 22.5)	21.8 (20.2, 23.5)	−1.1 (−3.5, 1.3)	0.37
Urine drug screen				
Positive for any substance	14 (24.1)	27 (45)	RR: 0.55 (0.32, 0.94)	0.029

Clinical Outcomes: Hospitalization rates were calculated as events per 100 person-months (PM) using negative binomial regression with person-time offset to account for varying follow-up durations. All-cause hospitalization rate includes any inpatient admission during the follow-up period. HF hospitalizations defined as admissions with primary or first secondary diagnosis of heart failure. Rate ratio was expressed as financial support vs control group.

Urine Drug Screen: Reported as risk ratio of financial support vs control group using modified Poisson regression with robust standard errors.

K10 = Kessler Psychological Distress Scale; PM = person-months; PSS = Perceived Stress Score; RR = risk ratio; other abbreviations as in Tables 2 and 3.

### Patient-reported outcomes and drug use

No significant between-group differences were observed for self-reported medication adherence (MMAS-8), perceived stress (PSS-10), or psychological distress (K10) at 1 month (Table 4). Participants in the financial support (versus control) group had significantly lower proportion of positive urine drug screens (24.1% vs 45.0%; risk ratio: 0.55 [0.32-0.94]; *P* = 0.029) at 1 month.

### Spending patterns and behaviors

Transaction data were obtained for 47 of the 76 participants in the financial support group (61.8%, Table 5). Cash withdrawals represented 40.0% of total intervention funds and were excluded from categorical analysis as they were not quantifiable by spending category. Expenditure patterns revealed that the majority of funds were directed toward essential living expenses rather than medications. Among traceable spending, retail and shopping represented the largest category (30.9%), followed by food and beverages (29.8%). Health care and pharmacy spending accounted for 5.8% of traceable expenditures. Among participants with baseline cost-related medication nonadherence (n = 35), spending patterns differed from those without (n = 12), with greater allocation toward health care and pharmacy (7.5% vs 0.0%; *P* = 0.038) (Table 5).

**Table 5:** Debit-Card Assessed Spending Patterns for Financial Support Arm at 1 Month

Spending Category	Percent of Traceable Spending		
	Overall (N = 47)	Cost-Related Nonadherence (n = 35)	No Cost-Related Nonadherence (n = 12)
Retail and shopping	30.9%	24.5%	53.4%
Food and beverage	29.8%	32.1%	21.5%
Transportation	9.3%	9.7%	7.7%
Convenience store	7.6%	7.8%	7.1%
Entertainment and leisure	5.9%	6.8%	2.8%
Healthcare and pharmacy	5.8%	7.5%	0.0%
Financial Services and fees	3.8%	4.9%	0.0%
Utilities and telecom	3.4%	4.0%	1.2%
Miscellaneous/other	2.2%	2.1%	2.8%
Bills and utility	1.3%	0.7%	3.4%

Analysis was restricted to only include spending within the first 30 days after randomization to financial support, only participants with transaction data including ≥$450 of added funds were included (n = 47, 61.8% of all financial support participants, 94.0% of financial support participants with captured transaction data). Cash withdrawals (40.0% of overall spending) were excluded from this analysis as these transactions were not quantifiable by spending category. Spending amounts were aggregated by category for each participant. Proportions calculated as percentage of total traceable spending after excluding cash withdrawals.

## Discussion

### Main findings

This article describes the trial design, socioeconomic eligibility framework, and baseline characteristics underpinning the FUND-HF pilot trial. The main study outcomes of this pilot trial are presented in the companion *JACC* report, whereas we have described the results of sensitivity and exploratory analysis in this article.^21^

### Context of prior financial support and adherence programs

Prior financial support programs have demonstrated mixed effects on health and well-being. The Baby’s First Years trial, which provided monthly payments of up to $333 to low-income mothers over 4 years, found no significant effects on child development or maternal well-being despite the substantial investment.^28^ A randomized lottery study in Chelsea, Massachusetts, demonstrated that providing up to $400 monthly for 9 months to low-income residents reduced emergency department utilization during the intervention period.^29^ However, both programs addressed poverty broadly through extended financial support. The present study extends the existing literature by evaluating a one-time financial support in a post-HF hospitalization population.

Although no prior studies have examined unrestricted cash transfers for a population defined by a cardiovascular condition, several related approaches have been evaluated. The MI-FREEE (Post-Myocardial Infarction Free Rx Event and Economic Evaluation) trial demonstrated that eliminating copayments for cardiovascular medications after myocardial infarction significantly improved medication adherence but did not reduce the primary clinical composite outcome.^17^ The ARTEMIS (Affordability and Real-world Antiplatelet Treatment Effectiveness After Myocardial Infarction Study) trial evaluated the use of medication vouchers to improve adherence to P2Y_12_ inhibitors after percutaneous coronary intervention and similarly found improvements in medication persistence with voucher-based approaches without difference in the primary clinical outcome.^18^ Behavior-based financial incentive trials have similarly demonstrated improvements in health behaviors, including doubled rates of antihypertensive medication adherence measured by electronic monitoring, although without corresponding blood pressure reduction, and increased physical activity among patients with or at high risk for atherosclerotic cardiovascular disease.^30^^,^^31^

Our study differs fundamentally from these approaches by providing unrestricted financial support rather than targeting specific domain or goal-based incentives, allowing participants to address whichever economic barriers were most pressing. Notably, unconditional financial support may offer practical advantages over conditional or lottery-based incentive approaches, given its simplicity of implementation and lower patient burden. More broadly, although recent policy initiatives, including those by the Centers for Medicare and Medicaid Services, have catalyzed screening and referral programs for unmet social needs, most health system responses remain downstream, such as offering medication vouchers, transportation assistance, or linking patients with food pantries once needs are identified.^20^^,^^32^^,^^33^ Our upstream approach represents a shift from health care system interventions toward direct adverse SDOH modification during critical clinical transitions.

### Mechanism of action considerations

The mechanism of improved adherence likely operates through alleviating broader economic stress in addition to directly subsidizing medication costs. Financial strain is independently associated with medication nonadherence even after adjusting for income,^34^ and patients experiencing financial difficulties engage in cost-reducing strategies such as skipping doses or delaying refills.^35^ However, the relationship extends beyond direct medication costs. Participants with 3 or more social risk factors including low income and high financial strain have more than 3-fold higher odds of medication nonadherence,^36^ and financial stress may reduce cognitive bandwidth available for health self-management behaviors.^37^ Consistent with this framework, the treatment effect of financial support on adherence to GDMT was greater among participants with higher baseline burden of adverse SDOH, supporting the hypothesis that the intervention operates primarily through alleviation of socioeconomic barriers to medication-taking behavior. Prior literature demonstrates cash transfers work through multiple pathways including alleviating financial barriers to health care access, reducing trade-offs between health needs and other necessities, and improving empowerment and agency.^38^ By providing unrestricted resources during the vulnerable postdischarge period, our intervention likely freed both economic and cognitive resources that could then be directed toward medication-taking behaviors. This interpretation is further supported by spending patterns in our study, in which participants allocated the majority of funds toward bills and essential expenses rather than medication or healthcare costs directly. Notably, participants reporting cost-related nonadherence at baseline directed greater spending toward health care–related expenses, suggesting that unrestricted financial support alleviated both competing economic demands broadly and enabled those facing specific medication cost barriers to address previously forgone needs.

### Interpretation of HRQOL findings

The lack of improvement in HRQOL among participants receiving financial support (vs usual care) likely reflects several factors. First, both study arms experienced substantial gains in KCCQ scores during the early postdischarge period, consistent with expected recovery trajectories following hospitalization for HFrEF, as observed in the PARADIGM-HF trial.^39^ Second, a 1 month follow-up may not have been sufficient to accrue differential HRQOL improvements or changes in disease severity markers resulting from improved medication adherence. Prior studies have demonstrated that the symptomatic and HRQOL benefits of therapies such as beta-blockers and mineralocorticoid receptor antagonists typically accrue gradually. This principle is supported by findings from the STRONG-HF (Safety, Tolerability and Efficacy of Rapid Optimization, Helped by NT-proBNP testinG, of Heart Failure Therapies) trial, in which the effects of high-intensity up-titration of GDMT on both clinical endpoints and QOL became more evident with greater follow-up duration from time of discharge, underscoring that the full impact of optimized therapy often requires longer follow-up to become apparent.^40^^,^^41^ Finally, HRQOL as assessed by the KCCQ-OSS encompasses multiple psychological, functional, and social dimensions that may require longer intervention periods to demonstrate measurable changes.^42^

### Adherence outcome selection considerations

TDM was selected as the key adherence outcome given its superior accuracy for detecting nonadherence. Prior studies have demonstrated that the MMAS-8 has limited sensitivity for detecting true nonadherence when compared against TDM, with 1 study of patients with treatment-resistant hypertension demonstrating the MMAS-8 identified only 26% of patients as low adherence while TDM revealed nonadherence in 51%.^43^ Prescription fill data, although commonly used in administrative database studies, do not confirm whether patients actually ingested dispensed medications and are impractical for assessing adherence over a short 4-week follow-up. Pill counts similarly overestimate adherence; in a study comparing return tablet counts against pharmacologic confirmation using plasma phenobarbital concentrations, 32% to 48% of patients deemed adherent by pill count had drug levels suggesting poor adherence.^44^ By contrast, serum-based TDM using liquid chromatography-tandem mass spectrometry provides direct biochemical confirmation of drug ingestion. However, for larger trials, complementary approaches such as electronic monitoring devices may enhance adherence assessment.

### Future research implications

Our pilot study findings can inform future larger trials powered for clinical endpoints. The observed treatment effect estimates for medication adherence provide preliminary data for sample size calculations in a definitive multicenter trial. Based on these pilot data, a larger multicenter RCT is being planned with more sustained financial support for up to 6 months, evaluating long-term (up to 12 months) clinical events, HRQOL, and medication adherence via TDM to determine whether longer-duration financial support can translate adherence improvements into meaningful clinical benefits.

In reflecting on the current pilot design, several alternative approaches merit consideration for future studies. Rather than a single lump-sum payment, more sustained periodic financial support (eg, monthly disbursements) may provide ongoing relief from competing economic demands and sustain improvement in adherence over a longer period. Alternatively, targeted medication support approaches could be compared with unrestricted financial support to determine whether medication-specific vs broad economic interventions are more effective. However, the spending data from this pilot trial showed that participants directed the majority of funds toward essential living expenses rather than medications. This highlights patient priorities with regard to their financial needs and suggests that addressing broader economic barriers may be important for this population. Future trials should also consider longer TDM follow-up at 6 and 12 months to determine the durability of adherence effects observed at 1 month.

### Strengths

This study demonstrates several notable strengths. We enrolled a socioeconomically disadvantaged HF population with high representation of racial/ethnic minorities and substantial burden of adverse SDOH, a population under-represented in contemporary clinical trials.^45^ Our cohort exhibited profound limitations in HRQOL at baseline (median KCCQ-OSS 39.1), consistent with prior studies which assessed KCCQ in the peridischarge period including REPORT-HF (International REgistry to assess medical Practice with lOngitudinal obseRvation for Treatment of Heart Failure) and REHAB-HF (A Trial of Rehabilitation Therapy in Older Acute Heart Failure Patients), reflecting both the timing of assessment (median 6 days postdischarge) and the substantial burden of adverse SDOH, which may have directly impaired HRQOL.^46^, ^47^, ^48^ The comprehensive socioeconomic phenotyping across multiple SDOH domains provided detailed characterization of this vulnerable population and led to enrollment of those most likely to benefit from a financial support intervention. In addition, the objective measurement of medication adherence through TDM eliminated bias inherent in self-reported adherence measures and revealed substantial differences that self-reported adherence, assessed via the MMAS-8 Scale, failed to detect. The significantly lower proportion of positive urine drug screens in the intervention group also demonstrated that financial support did not enable inappropriate substance use, addressing potential safety concerns about financial support interventions.

### Study limitations

However, several limitations merit consideration. The single-center design in a specific urban safety-net environment may limit generalizability to other settings and populations. The Parkland health system provides medications through its own pharmacy system at reduced cost, and the socioeconomic profile of this cohort may differ from HF populations in other health care settings. Larger multicenter trials are needed to establish generalizability. The 1-month follow-up period precluded assessment of longer-term clinical outcomes and durability of the adherence effect. The intervention amount of $500, while representing 83% of participants’ median monthly income, was determined pragmatically, leaving uncertainty about optimal financial support parameters. TDM-based adherence is limited by the availability of validated assays, and assays for key HF therapies including sacubitril/valsartan and SGLT2 inhibitors were not available. For larger, more scalable trials, alternative adherence approaches such as electronic monitoring devices or alternative patient-reported adherence surveys may be more practical. The potential for a Hawthorne effect should also be considered, as participants were aware they would undergo TDM and urine toxicology screening at the follow-up visit as part of the consenting process which may have influenced their behavior in both study arms; however, the presence of a concurrent control group exposed to the same monitoring mitigates this concern, and longer follow-up in future studies would reduce the influence of any such short-term behavioral modification. Finally, missing data due to loss to follow-up (8.5% overall) should be considered, although sensitivity analyses using reference-based imputation under conservative assumptions yielded results consistent with the primary analysis.

## Conclusions

The FUND-HF pilot trial demonstrates the feasibility of identifying, enrolling, and delivering a postdischarge financial support intervention to socioeconomically vulnerable patients with HFrEF. The comprehensive socioeconomic eligibility framework and detailed SDOH phenotyping characterized a population with profound economic vulnerability, severely impaired HRQOL, and high burden of adverse SDOH. Together with the companion clinical outcomes report, these data provide preliminary treatment effect estimates, safety data, and key design parameters to inform a larger, multicenter trial evaluating whether financial support can improve postdischarge outcomes in socioeconomically disadvantaged HF populations.

## Funding support and author disclosures

Internal research support from 10.13039/100007914the University of Texas Southwestern Medical Center to Drs Peterson and Pandey. Dr Keshvani has received consultant fees from Tricog Health, 10.13039/501100016198Idorsia Pharmaceuticals, and Science37. Dr Segar has received honoraria from 10.13039/501100016198Idorsia Pharmaceuticals and Otsuka; nonfinancial support from 10.13039/100004334Merck; is an advisor for AccurKardia; is on the advisory board of descendantsDNA; and is the founder of ReCODE Medical. Dr Peterson has received grant funding from Amgen; and serves on study advisory boards for Janssen and Novo Nordisk. Dr Pandey has received grant funding outside the present study from National Institute of Health, American Heart Association, Roche, Ultromics, Bayer, AstraZeneca, Applied Therapeutics and 10.13039/100005564Gilead Sciences; has received honoraria outside the present study as an advisor/consultant for Tricog Health Inc, Lilly Rivus, Roche Diagnostics, Axon Therapies, Edward Lifesciences, Science37, Novo Nordisk, Bayer, Medical AI, Astra Zeneca, Baylor Scott and White Research Institute, Boehringer Ingelheim, iRhythm Technologies, Tourmaline Bio, Merck, Sarfez Pharmaceuticals, Ultromics, Kardigan, Tenax Pharma, Alnylam, Abbott, Kilele Health, Anumana, Acorai, Novartis, and Antlia Biosciences. All other authors have reported that they have no relationships relevant to the contents of this paper to disclose.
