# Supplementary material for: Design, Characteristics, and Implementation of the Financial Support for Low-Income Patients with Heart Failure Trial
Source: JACC Adv. 2026 Mar 16;5(5):102698. doi: 10.1016/j.jacadv.2026.102698 (PMC13221884; doi:10.1016/j.jacadv.2026.102698)

**Supplemental Table 1:** Questionnaires and Assessments Used to Determine Eligibility

| **Domain of Social Need** | **Validated Instrument Used** | **Eligibility Criteria** |
| --- | --- | --- |
| **Employment Security** | Job Security section, General Social Survey | Unemployment defined as unemployed or retired |
| **Housing Stability** | Self-report survey | Self-reported concern about current or future housing |
| **Household Crowding** | Self-report of household composition | Household crowding defined as ratio of household residents to rooms >1 |
| **Medication Adherence** | Medicare Current Beneficiary Survey (MCBS), Cost-related items | At least one affirmative response to cost-related medication nonadherence questions |
| **Food Security** | U.S. Household Food Security Survey Module: Six-Item Short Form | Food insecurity defined as raw score ≥2 |
| **Transportation Barriers** | Transportation Barriers Measure | Self-reported difficulty with transportation to access medical care |
| **Social Support** | ENRICHD Social Support Instrument | Low social support defined as total ESSI score <18 |
| **Interpersonal Violence** | Hurt Insult Threaten Scream (HITS) screening tool | Interpersonal violence defined as total HITS score ≥10 |
| **Discrimination** | Everyday Discrimination Scale and Major Experiences of Discrimination Questionnaire | At least one affirmative response indicating experience of discrimination |
| **Abbreviations:** ESSI, ENRICHD Social Support Instrument; HITS, Hurt Insult Threaten Scream; MCBS, Medicare Current Beneficiary Survey; SAHL, Short Assessment of Health Literacy. | | |

**Supplemental Table 2:** Baseline Characteristics of Participants Who Completed Versus Did Not Complete 1-Month Follow-Up

| **Variable** | **Completed Follow-Up (n=140)** | **Did Not Complete Follow-Up (n=13)** |
| --- | --- | --- |
| Age, years | 53.0 (10.9) | 50.2 (11.9) |
| Male sex | 106 (75.7) | 10 (76.9) |
| Race/Ethnicity |  |  |
| Non-Hispanic White | 16 (11.4) | 0 (0.0) |
| Hispanic White | 19 (13.6) | 4 (30.8) |
| Black | 102 (72.9) | 9 (69.2) |
| Other | 3 (2.1) | 0 (0.0) |
| County Indigent Health Program | 51 (36.4) | 3 (23.1) |
| Insurance |  |  |
| Uninsured With County Indigent Support | 42 (30.0) | 3 (23.1) |
| Uninsured Without County Indigent Support | 33 (23.6) | 5 (38.5) |
| Medicare/Medicaid/Dual Eligible | 44 (31.4) | 4 (30.8) |
| Marketplace/Private | 21 (15.0) | 1 (7.7) |
|  |  |  |
| *Clinical Characteristics* | | |
| Hypertension | 126 (90.0) | 12 (92.3) |
| Diabetes | 55 (39.3) | 6 (46.2) |
| Hyperlipidemia | 74 (52.9) | 7 (53.8) |
| CVA/TIA | 16 (11.4) | 3 (23.1) |
| Coronary Artery Disease | 41 (29.3) | 3 (23.1) |
| Mental Health Disease | 26 (18.6) | 2 (15.4) |
| Atrial Fibrillation/Flutter | 53 (37.9) | 5 (38.5) |
| Substance Use Disorder | 25 (17.9) | 6 (46.2) |
| NYHA Class III | 62 (44.3) | 9 (69.2) |
| Non-Ischemic HF Etiology | 114 (81.4) | 10 (76.9) |
| New HF diagnosis | 15 (10.7) | 2 (15.4) |
| LVEF | 25.0 [20.0, 33.3] | 27.0 [20.0, 33.0] |
| Implantable Cardioverter Defibrillator | 42 (30.0) | 4 (30.8) |
| BMI, kg/m^2^ | 30.5 [25.6, 40.5] | 30.7 [27.2, 36.0] |
| SBP, mmHg | 120.0 [107.3, 137.0] | 112.7 [105.3, 127.3] |
| Discharge eGFR, mL/min/1.73m^2^ | 60.3 (19.4) | 55.7 (29.6) |
| Admission NT-proBNP, pg/mL | 3638.0 [2208.0, 7357.0] | 5150.00 [2314.8, 8260.3] |
| *Social Needs and Eligibility* | | |
| Days from Randomization to Discharge | 6.0 [4.0, 8.0] | 3.0 [2.0, 6.0] |
| Self-Reported Monthly Income, $ | 575.0 [0.0, 1149.5] | 833.0 [0.0, 1300.0] |
| Self-Reported Monthly Income of $0 | 55 (39.3) | 5 (38.5) |
| Domains of Social Need* |  |  |
| Unemployment | 115 (82.1) | 9 (69.2) |
| Housing Instability | 69 (49.3) | 7 (53.8) |
| Household Crowding | 38 (27.1) | 3 (23.1) |
| Cost-Related Medication Non-Adherence | 106 (75.7) | 10 (76.9) |
| Food Insecurity | 117 (83.6) | 13 (100.0) |
| Transportation Barriers | 92 (65.7) | 7 (53.8) |
| Low Social Support | 63 (45.0) | 6 (46.2) |
| Interpersonal Violence | 13 (9.3) | 2 (15.4) |
| History of Major Discrimination | 109 (77.9) | 10 (76.9) |
| Total | 5.0 [4.0, 7.0] | 5.0 [5.0, 6.0] |
| *HF Medication Utilization* | | |
| Beta Blocker | 122 (87.1) | 9 (69.2) |
| RASi | 122 (87.1) | 11 (84.6) |
| MRA | 116 (82.9) | 10 (76.9) |
| SGLT2i | 115 (82.1) | 8 (61.5) |
| Quadruple GDMT | 83 (59.3) | 5 (38.5) |
| Metoprolol | 83 (59.3) | 8 (61.5) |
| Spironolactone | 113 (80.7) | 10 (76.9) |
| Furosemide | 64 (45.7) | 4 (30.8) |
| Amlodipine | 7 (5.0) | 1 (7.7) |
| Hydralazine | 11 (7.9) | 1 (7.7) |
| Nifedipine | 0 (0.0) | 0 (0.0) |
| Prazosin | 1 (0.7) | 0 (0.0) |
| Number of Medications Amenable to TDM per participant | 2.0 [1.00, 2.3] | 2.0 [1.0, 2.0] |
| Prescribed a Testable GDMT (metoprolol or spironolactone) | 130 (93.5) | 13 (100) |
| *Questionnaires* | | |
| KCCQ-OSS, numeric | 39.3 [24.8, 54.4] | 31.3 [12.0, 70.3] |
| MMAS-8, numeric | 5.8 [4.0, 7.0] | 4.8 [3.8, 7.0] |
| MMAS-8, categorical |  |  |
| Low Adherence | 82 (58.6) | 7 (53.8) |
| Medium/High Adherence | 58 (41.4) | 6 (46.2) |
| Self-Reported Spending, $ |  |  |
| Healthcare | 1.0 [0.00, 85.5] | 10.0 [0.0, 65.00] |
| Bills/Essentials | 450.0 [182.5, 838.5] | 410.0 [120.0, 820.0] |
| Miscellaneous | 90.0 [0.0, 215.0] | 40.0 [0.0, 110.0] |
| Values shown are n(%), mean(SD), or median[IQR]  *Domains of Social Need assessed using the following: unemployment defined as unemployed or retired, housing stability defined as self-reported concern about current or future housing, household crowding defined as ratio of residents to rooms >1, cost-related medication adherence as at least one affirmative answer to the four cost-related medication nonadherence questions adapted from the Medicare Current Beneficiary Survey (MCBS); food insecurity defined as raw score ≥ 2 using the U.S. Household Food Security Survey Module: Six-Item Short Form; transportation barriers defined as self-reported difficulty with transportation to access medical care; low social support defined as <18 on ENRICHD Social Support Instrument; interpersonal violence defined as ≥10 on Hurt Insult Threaten Scream (HITS) screening tool  Low adherence defined as MMAS-8 score <6; Medium adherence = 6 to <8; High adherence = 8.  Abbreviations: BMI - Body Mass Index; CVA/TIA - Cerebrovascular Accident/Transient Ischemic Attack; eGFR - Estimated Glomerular Filtration Rate; IQR - Interquartile Range; KCCQ-OSS - Kansas City Cardiomyopathy Questionnaire Overall Summary Score; LVEF - Left Ventricular Ejection Fraction; MMAS-8 - Morisky Medication Adherence Scale, 8-item; MRA - Mineralocorticoid Receptor Antagonist; NT-proBNP - N-terminal pro B-type Natriuretic Peptide; NYHA - New York Heart Association; RASi - Renin-Angiotensin System Inhibitor; SBP - Systolic Blood Pressure; SD - Standard Deviation; SGLT2i - Sodium-Glucose Cotransporter-2 Inhibitor. | | |

**Supplemental Table 3: Treatment Effect Modification by Baseline Income and SDOH Burden**

| **Outcome** | **Below Median** | | **Above Median** | | **P_interaction_** |
| --- | --- | --- | --- | --- | --- |
|  | **Difference**  **(95% CI)** | **P Value** | **Difference**  **(95% CI)** | **P Value** |  |
| ***By SDOH Burden*** | | | | | |
| *Medication Adherence* |  |  |  |  |  |
| Proportional Adherence to all medications, EMM (95% CI) | 0.09 (-0.06, 0.24) | 0.24 | 0.35 (0.17, 0.53) | <0.001 | 0.053 |
| Proportional Adherence to GDMT, EMM (95% CI) | 0.06 (-0.11, 0.23) | 0.47 | 0.42 (0.23, 0.60) | <0.001 | 0.016 |
| *Quality of Life* |  |  |  |  |  |
| KCCQ-OSS | 2.5 (-6.3, 11.4) | 0.57 | -2.1 (-12.1, 7.8) | 0.67 | 0.51 |
| ***By Baseline Income*** | | | | | |
| *Medication Adherence* |  |  |  |  |  |
| Proportional Adherence to all medications, EMM (95% CI) | 0.22 (0.05, 0.39) | 0.012 | 0.19 (0.02, 0.35) | 0.025 | 0.78 |
| Proportional Adherence to GDMT, EMM (95% CI) | 0.23 (0.05, 0.39) | 0.014 | 0.20 (0.02, 0.38) | 0.027 | 0.76 |
| *Quality of Life* |  |  |  |  |  |
| KCCQ-OSS | -3.7 (-13.5, 6.1) | 0.46 | 3.2 (-6.3, 12.6) | 0.51 | 0.29 |
| Median baseline monthly income was $600. Median SDOH burden was 5 domains (of 9 assessed); participants were stratified as ≤5 domains (below/at median) and >5 domains (above median). Treatment effect modification assessed using interaction terms between treatment assignment and each subgroup variable. Income evaluated using log-transformed values.  Proportional adherence by TDM calculated as estimated marginal means from binomial generalized linear models, expressed as absolute differences in proportions (0 to 1). GDMT adherence outcomes restricted to metoprolol and spironolactone. KCCQ-OSS models adjusted for baseline KCCQ-OSS.  Abbreviations: CI, confidence interval; GDMT, guideline-directed medical therapy; KCCQ-OSS, Kansas City Cardiomyopathy Questionnaire Overall Summary Score; SDOH, social determinants of health; TDM, therapeutic drug monitoring. | | | | | |

**Supplemental Table 4:** Outpatient Follow-up Visits at 1-Month

| **Visit Type** | **Financial Support (n=76)** | **Control (n=77)** | **Difference/OR (95% CI)** | **P-value** |
| --- | --- | --- | --- | --- |
| *Transitional Care Program Visits* | | | | |
| Percent of visits completed, mean (SD) | 51% (48) | 64% (47) | -0.13 (-0.35, 0.10) | 0.26 |
| All visits attended, n (%) | 17 (45.9%) | 22 (61.1%) | 0.54 (0.21, 1.37) | 0.20 |
| No visits attended, n (%) | 16 (43.2%) | 12 (33.3%) | 1.52 (0.59, 3.94) | 0.39 |
| *Cardiology Visits* | | | | |
| Percent of visits completed, mean (SD) | 62% (48) | 57% (47) | 0.05 (-0.19, 0.28) | 0.68 |
| All visits attended, n (%) | 19 (59.4%) | 17 (51.5%) | 1.38 (0.52, 3.67) | 0.52 |
| No visits attended, n (%) | 11 (34.4%) | 12 (36.4%) | 0.92 (0.33, 2.54) | 0.87 |
| *Other Specialty Visits* | | | | |
| Percent of visits completed, mean (SD) | 74% (41) | 50% (44) | 0.24 (-0.02, 0.50) | 0.07 |
| All visits attended, n (%) | 16 (64.0%) | 7 (36.8%) | 3.05 (0.88, 10.52) | 0.08 |
| No visits attended, n (%) | 5 (20.0%) | 7 (36.8%) | 0.43 (0.11, 1.66) | 0.22 |
| *Primary Care Visits* | | | | |
| Percent of visits completed, mean (SD) | 59% (49) | 71% (45) | -0.12 (-0.53, 0.29) | 0.56 |
| All visits attended, n (%) | 6 (54.5%) | 8 (66.7%) | 0.60 (0.11, 3.24) | 0.55 |
| No visits attended, n (%) | 4 (36.4%) | 3 (25.0%) | 1.71 (0.29, 10.30) | 0.56 |
| *Total Follow-up* | | | | |
| Percent of visits completed, mean (SD) | 60% (42) | 57% (43) | 0.03 (-0.12, 0.18) | 0.72 |
| All visits attended, n (%) | 30 (44.1%) | 25 (41.0%) | 1.14 (0.56, 2.29) | 0.72 |
| No visits attended, n (%) | 19 (27.9%) | 18 (29.5%) | 0.93 (0.43, 1.99) | 0.84 |
| Data presented as n (%) for binary outcomes and mean (SD) for continuous outcomes expressed as percentages. Binary outcomes analyzed using logistic regression with odds ratios (95% CI). Continuous outcomes analyzed using linear regression with mean differences (95% CI). Sample sizes vary by visit type based on participants with scheduled appointments in each category. | | | | |

**Supplemental Figure 1:** Distribution of Detectable Serum Drug Concentrations by Medication and Treatment Group

Caption: Boxplots depict the distribution of serum drug concentrations among participants by treatment group for each tested medication. Sample sizes vary by drug and treatment group and are shown above each panel.


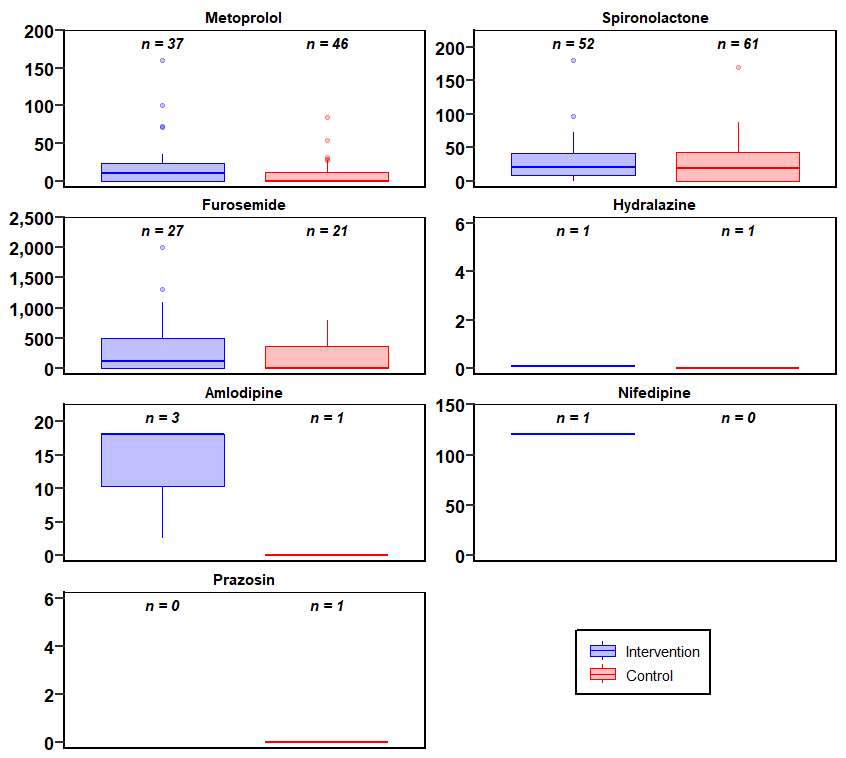

Supplement: Supplemental Material [file mmc1.docx]
